# The Scabies Problem on Active Service

**Published:** 1917-11

**Authors:** 


***The Scabies Problem on Active Service.*** By H. Mac Cormac, Major R. A. M. C. (T. C.). Abstrated from the *British Medical Journal*, Sept. 22, 1917.

The author believes that treatment for scabies should receive more consideration from army workers, because it is next to pediculosis the most prevalent skin disease, and because when complicated, as is frequently the case, the period of cure is on an average about 31 days, and therefore constitutes a serious loss of service.

Unlike usual scabies, the army variety rarely shows burrows or other lesions on the hands, and is often accompanied with pyodermic complications. The eruption, however, is distributed as usual. Vesicles are more common. It may often be distinguished from pediculosis by papules or crusts on either the skin or the mucous membrane of the penis.

Complications like impetigo, boils, dermatitis, or inguinal adenitis, may conceal the presence of scabies. Since such cases require much longer treatment (about ten times as long) they must be very carefully diagnosed. Special care should be exercised to distinguish it from pediculosis (in which there is usually no infection of the hands, wrists, and penis); from pompholyx (in which vesicular lesions are deeper, more numerous, more difficult to break, and usually confined to the feet); from venereal disease (with which it is more easily confused than might be supposed); and from papular urticaria (which shows small red papules all over the body, especially the abdomen and anterior axillary folds, and which does not appear on the hands, wrists, or penis).

Prevention requires regular inspection, to detect " carriers " and to segregate cases. Even horses should be inspected. Blankets are considered a chief source, and should be frequently sterilized.

The treatment recommended instead of sulphur vapor (which is considered harmful to the patient, uncertain in effect, and likely to produce a class of scabies " carriers ") is inunction with sulphur ointment applied with utmost thoroughness and care. It should be preceded by a thorough soaping and steeping for fifteen minutes in a warm bath to the neck. When possible the skin should be thoroughly rubbed with a nail brush to open burrows and vesicles. If all are not opened in this fashion a surgical needle should be used. References are given.

*Le Gérant :* O. PORÉE. Paris — imp. LAHURE, 9, rue de Fleurus.

